# Evidence from a Systematic Review and Meta-Analysis Pointing to the Antidiabetic Effect of Polyphenol-Rich Plant Extracts from *Gymnema montanum*, *Momordica charantia* and *Moringa oleifera*

**DOI:** 10.3390/cimb44020049

**Published:** 2022-01-28

**Authors:** Michal Krawczyk, Izabela Burzynska-Pedziwiatr, Lucyna Alicja Wozniak, Malgorzata Bukowiecka-Matusiak

**Affiliations:** Chair of Medical Biology, Laboratory of Metabolomic Studies, Department of Structural Biology, Faculty of Medicine, Faculty of Biomedical Sciences, Medical University of Lodz, Zeligowskiego 7/9, 90-752 Lodz, Poland; michal.s.krawczyk@gmail.com (M.K.); izabela.burzynska-pedziwiatr@umed.lodz.pl (I.B.-P.); lucyna.wozniak@umed.lodz.pl (L.A.W.)

**Keywords:** diabetic rats, plant extracts, phytochemicals, supplementation, oxidative stress

## Abstract

In vitro and animal model studies are of great interest for selecting new phytochemicals, including polyphenols with antioxidative properties, as candidates for antidiabetic drugs. This review provides evidence from a critical literature data analysis on the effects of plant extract supplementation in diabetes mellitus management. We considered and meta-analyzed the efficacy of oral supplementation of plant extracts in animal model studies and examined physiological and oxidative stress parameters. Finally, 23 articles were included in the meta-analysis, revealing three plants with experimentally confirmed in vivo and in vitro antidiabetic properties: *Gymnema montanum*, *Momordica charantia* and *Moringa oleifera.* The following parameter changes resulted from an investigation of the supplementation: reduced oxidative stress, decreased insulin resistance, increased insulin release, reduced adiposity, and a modulatory effect on glycolysis and gluconeogenesis, as well as attenuation of diabetes-associated weight loss, reduced fasting blood glucose and lowered oxidative status. A comparison of *Gymnema montanum* versus Glybenclamide revealed the superiority of extracts over drug administration in some aspects. Although the analyzed extracts are promising candidates for antidiabetic treatment, there is much inconsistent data in the literature. Therefore, ultimate references for using these compounds in the prevention of diabetes are currently not applicable.

## 1. Introduction

According to the WHO, diabetes mellitus (DM) is one of the most widespread chronic diseases, and the number of cases is rising rapidly. The number of affected patients in 2014 reached 422 million, an almost two-fold increase compared to 1980 [1]. Current estimations predict that diabetic patients will reach 578 million by 2030 and 700 million by 2045 [2].

Oxidative stress (OS) is one of the leading causes of the development of diabetes and its complications [3]. Although organisms have an integrated antioxidant defense system to block the negative impact of reactive oxygen species (ROS), diabetes can cause this system to fail. Hence, supplementation with exogenous plant-derived antioxidants might possess capacities to avert oxidative stress-induced diseases.

Recently, there have been numerous studies in which plant extracts were used to treat various diseases with traditional medicines [4]. It is estimated that nearly a quarter of all modern medicines are derived from natural products [5]. Among the renowned antioxidant properties of several plants––including green tea, cinnamon, curcumin, grape seeds, and many berries––several new species used in traditional medicine have been documented. In vitro and animal model studies reflect an interest in selecting new phytochemical resources that possess antioxidative properties as candidates for drugs in antidiabetic approaches.

On the one hand, our long-term interest as a team is focused on investigating the molecular etiology of DM, in particular gestational diabetes mellitus (GDM) [6]; on the other hand, we are investigating the potential application of phytochemicals with antioxidant/antidiabetic activity obtained from the defatted seeds of *Oenothera paradoxa*. During our search for new sources of phytochemicals applicable to the management of DM, as revealed in animal model studies, we recently performed a meta-analysis to verify the efficacy of oral supplementation with plant extracts. We selected *Gymnema montanum*, *Momordica charantia* and *Moringa oleifera* since the well-documented data for DM-induced rats revealed their potential contribution to diabetes management and provoked more detailed studies.

### 1.1. Gymnema montanum Effect on Diabetes

*Gymnema montanum* (*G. montanum*, GM) is an endemic, woody climbing shrub found mainly in Africa and India. Leaves of GM have medical applications, andthey have a long history of use in India’s Ayurvedic medicine as an antidiabetic drug, diuretic, and digestive stimulant [7].

Currently, extracts from *Gymnema* leaves have found application in metabolic syndrome, weight loss, and cough.

Ananthan et al. studied the effect of the ethanolic extract from the leaves of *Gymnema montanum* on lipid-peroxidation-induced oxidative stress in experimental diabetes animal models [8]. Male Winstar rats were used, and diabetes was induced with alloxan monohydrate. One group of rats was treated with the plant extract, and the second with the antidiabetic drug Glibenclamide. The plasma levels of thiobarbituric acid reactive substances (TBARS) and hydroperoxides were estimated.

Furthermore, glutathione (GSH) and Vitamin C were investigated [9]. The studies confirmed increased lipid peroxidation in the plasma of diabetic rats. The study by Ananthan et al. showed that the plasma level of peroxides in rats fed with *Gymnema montanum* extract tended to be near the average. Further studies showed that the investigated plant also had an impact on the appropriate level of natural antioxidants such as Vitamin C. Several studies reported decreased levels of Vitamin C and Vitamin E among diabetic patients [10,11]. However, supplementation with *Gymnema montanum* extract increased these crucial antioxidant vitamins [12].

Ananthan et al. [8] also concluded that the administration of the analyzed extract to the diabetic rats caused increased GSH. The authors suggested that this increase activates enzymes needed for the appropriate action of GSH, such as glutathione peroxidase and glutathione S-transferase. They also hypothesized that the antidiabetic effect of *Gymnema montanum* extract and its ability to decrease the blood glucose level could be due to a strengthened insulin release. Furthermore, the efficacy of the plant depends on the degree of β-cell destruction. Another essential feature of *Gymnema montanum* connected with the antioxidative properties of this plant is its ability to inhibit lipid peroxidation 

Diabetes is a disorder with many complications, one of which is abnormalities in the lipid profile. A disturbances in lipids metabolism after glucose impairment is one of the significant pathogenesis factors. It is connected mainly with the enhanced use of fatty acids from adipose tissue, and second with the increase of free fatty acids in the blood [13]. Impairments in lipids metabolism are connected with insulin deficiency and could cause hypercholesterolemia and hypertriglyceridemia [14]. Furthermore, there are pieces of evidence reporting that diabetes could change the lipid profile, which can impact cellular processes such as ion permeability and receptor interactions [15,16,17]. Ramkumar et al. studied the effects of *Gymnema montanum* on the fatty-acid composition and lipid-profile level in diabetic rats. They also used Glibenclamide as a reference drug [18]. In the alloxan-induced diabetic rats, the levels of glucose and lipids such as total cholesterol (TC), low-density lipoprotein (LDL) and triglycerides (TG), both in serum and in selected tissues, were increased. However, after administering *Gymnema montanum* extract, a significant decrease in blood glucose and lipid content was observed. The same study carried out using Glibenclamide showed that the modulatory effect of *Gymnema montanum* was better than the traditionally used drug. Worth underlining is that treating non-diabetes rats with the same concentration of GM extract did not cause any adverse effects. The authors postulated two potential antihyperlipidemic mechanisms. One was the insulin stimulatory effect of GM on β-cells, and the second was the insulin-mimetic activity of GM, which improved the movement of glucose from plasma to peripheral tissues. Since no adverse effects among the non-diabetic rats was observed, the first of the proposed mechanisms was probably more likely.

It was observed [19] in diabetic rats that the level of monounsaturated fatty acids, especially oleic acid, increased significantly, while polyunsaturated fatty-acid (PUFA) levels mainly linolenic and arachidonic acids, decreased. This could have been associated with the susceptibility of PUFAs to the action of free radicals due to the presence of double bonds. The synthetic pathway of the PUFAs requires two essential steps: desaturation and elongation. Diabetes lowers the rate-limiting desaturation step, especially the activity of δ-6-desaturase, which is the enzyme responsible for conversion of linoleic acid to γ-linolenic acid and α-linolenic acid to stearidonic acid [20]. It is known that PUFAs decrease thrombosis and atherosclerosis, and in this way they lower the probability of cardiovascular disorders [21]. The *Gymnema montanum* extract supplementation reverses changes in lipid content and helps maintain the proper composition of tissue fatty acids.

The extract studied by Ramkumar et al. was analyzed by gas chromatography coupled with mass spectrometry, and it was found that it contained 11.57% *w/w* of carvacrol, 6.77% of erythritol,4.58% of gallic acid and 3.09% of quercetin [21].

It is postulated that phytochemicals present in GM extract, particularly gallic acid, resveratrol and quercetin, possess the antioxidative, antidiabetic and antihyperlipidemic properties [22] that play a pivotal role in lowering blood glucose in diabetic patients and in improving the action of insulin.

### 1.2. Momordica charantia Effect on Diabetes

*Momordica charantia* (*M. charantia*, MC), a plant belonging to the Cucurbitaceae family, is commonly known as a bitter gourd, balsam pear, bitter melon, or Karela. All plant parts have a bitter taste, including the fruit. India, Japan, Singapore, Vietnam, Amazon, East Africa, Brazil, Malaya, China, Thailand, Colombia, Cuba, Ghana, Haiti, India, Mexico, New Zealand, Nicaragua, Panama, the Middle East, Central and South America are the regions where MC is cultivated. At maturation, the fruit of *M. charantia* can be used as a dietary food, and because of its multiple beneficial activities, has also been used as a herbal medicine [23].

In ancient history, the seed and fruit were used as medication for diabetes. The other fractions of *M. charantia*––roots, leaves and even vines––have been used in folk medicine to treat other diseases like diarrhea, toothache and furuncle. Therefore, this plant is the subject of many ongoing studies investigating its potential in preventing and treatingseveral diseases. Each year, more and more papers reveal the plausible effects of supplementation with *M. charantia*, thereby strongly indicating that this plant possesses various pharmacological functions: antidiabetic, anthelmintic, abortifacient, antimalarial, antimutagenic, antilipolytic, antifertility, hepatoprotective, anti-inflammatory, contraceptive and laxative, anti-ulcerogenic, antioxidative and immune-modulatory [24].

A more comprehensive application of *M. charantia* in multiple areas of medicine is still restricted due to adverse effects observed in many studies. Some of these are hypoglycemic coma in children, and toxicity or even death in laboratory animals [23].

Extracts of this plant consist of a broad-spectral resource of phytoconstituents. These compounds are usually divided into the following main groups: carbohydrates, polysaccharides, proteins and peptides, lipids, terpenoids, saponins, phenolics and sterols. Despite these main groups, compounds such as unsaturated fatty acids, alkaloids, amino acids, minerals and vitamins are also contained in *M. charantia*. The distribution of specific compounds varies among different plant parts [25].

Gallic acid, protocatechuic acid, gentisic acid, (+)-catechin, vanillic acid, syringic acid, (−)-epicatechin, p-coumaric acid, benzoic acid, sinapinic acid, o-coumaric acid, chlorogenic acid, t-cinnamic acid and t-ferulic acid are the most abundant flavonoids and phenolic compounds in *M. charantia*. Their concentration varies in the range of 1.77 ± 0.72% [26], and the fruit is considered to be the major source of phenolic components. It has been proven that an increase in flavonoid concentration is linearly correlated with an increase in antioxidant capacity of *M. charantia*, probably due to the fact that flavonoids are one of the most effective free radical scavengers and antioxidants [27].

Quinic acid and catechin were determined to be the most abundant flavonoids along with gallic acid, gentisic acid, chlorogenic acid, and epicatechin. Different phenolic acid constituents revealed different distributions among various plant parts. An investigation of extracts derived from different plant parts also revealed the presence of protocatechuic acid, p-coumaric acid, syringic acid, vanillic acid and benzoic acid, tannic acid, ferulic acid, and caffeic acid [28].

Ethanol extract from the dried powder of *Momordica charantia* was evaluated by Arafatet al. as an antidiabetic treatment in alloxan-induced diabetes rats [29]. Alloxan administration caused hyperglycemia in the tested animals. The HPLC quantitative analysis of the extract showed a high amount of ellagic acid (307.78 mg/100g of dry extract) and myricetin (180.6 mg/100g of dry extract). The concentration of gallic acid was also relatively high—extract supplementation to the diabetic rats’ effects in serum glucose decreased. Several shreds of evidence reported that ellagic acid and myricetin might prevent hepatic steatosis and fibrosis in diabetic patients by improving Nrf2 and CPT1 as well as decreasing protein levels of NF-κB [30,31]. These results were also confirmed by studies carried out by Arafat [29]. *Momordica charantia* supplementation of diabetic rats prevented mononuclear cell infiltration and decreased myeloperoxidase activity.

Furthermore, this effect may be facilitated partly by inhibiting oxidative stress and inflammation in the liver by the phenolic components of the MC extract. Some researchers indicated that MC extract enhanced insulin secretion by stimulating β-cells [32,33]. On the other hand, Mahomoodally et al. [34] suggested that the administration of MC extract in lab animals reduced glucose absorption from gut walls and stimulated glucose uptake and use in adipose muscle tissue.

It is known that inflammation and oxidative stress are the main factors responsible for liver damage in diabetic patients [35]. Arafat et al. [28] observed decreased concentration in all antioxidant enzymes in alloxan-induced diabetic rats. However, animals treated with the *Momordica charantia* ethanolic extract significantly enhanced all antioxidant parameters, including improvement of the GSH concentration, SOD and CAT activities. *M. charantia* polyphenols possess anti-inflammatory and antioxidative properties that play a crucial role in the inhibition of DM and the progression of its complications, mainly due to the inhibition of the accumulation of advanced glycation end-products (AGE’s), which were inhibited significantly by tannins as identified in the extracts. In addition to reducing AGE’s accumulation, tannins also take part in inflammatory state regression and insulin resistance reduction. As a result of the latter two, micro- and macrovascular complications of DM decreased; thus, the analyzed extracts revealed the potential for preventing vascular complications [36]. The correlation between the phenolic acid concentration of *M. charantia* fruit extract and antiglycation activity was assessed and found to be linearly interconnected with increased suppression of the glycation process [37].

Based on the abovementioned activities of particular chemical components of *M. charantia* extract and broad literature data, it can be reasserted that, in correlation with its composition, the MC extracts possess the following general activities: antidiabetic, antioxidant, antiviral, antimicrobial, anthelmintic, abortifacient, antimalarial, antimutagenic, antilipolytic, antifertility, hepatoprotective, anti-inflammatory, antitumor, hypolipidemic, immunomodulatory, and wound healing.

### 1.3. Moringa oleifera Effects on Diabetes

*Moringa oleifera* (*M. oleifera*, MO) Lam is a plant that belongs to the *Moringaceae* family and naturally occurs widely in many tropical and subtropical areas [38,39]. This plant originates from the western and sub-Himalayan tracts, India, Pakistan, Asia Minor, Africa and Arabia [40,41]. It is well-known as the “drumstick tree” or “the horseradish tree” based on the taste of ground root preparations and the ben oil tree from seed-derived oils [41]. Diverse parts of *M. oleifera* (leaves, fruits, flowers, and roots) are commonly used as food, nutraceuticals, traditional medicine, water sanitization, and biofuel production due to their rich source of many vital nutrients bioactive compounds [42]. 

Mounting evidence reports that *M. oleifera* parts, especially the leaves, have nutritional properties or can be used in diet supplementation [43]. Using *M. oleifera* extract in food products has improved overall nutritional quality, sensory properties and shelf life. The use of leaves, seed and flower powder is well known in various food applications, such as in fortifying amala (stiff dough), ogi (maize gruel), bread, biscuits, yoghurt, cheese, and soups [42].

*Moringa* in non-traditional medicine is known for treating many diseases, including diabetes, cancer, cardiovascular, neurological, gastroenterological, and inflammatory disorders. *Moringa oleifera* leaves are the most commonly used part of this plant and contain beta-carotene, vitamins B, C, E, minerals, polyphenols [44,45,46], oxidase, catalase, alkaloids, glucosinolates, isothiocyanates, tannins, and saponins [38,47]. In *Moringa* seeds, niazimicin and niazirin and a rhamnosyl benzyl carbamate, rhamnosyl benzyl isothiocyanate, and various derivatives of β-sitosterol were identified. This plant’s stems, roots, and other morphological parts are not well researched, unlike the leaves and seeds; therefore, the data on the composition is relatively limited [47].

The biological activity of *M. oleifera* is usually linked to the presence of phenolic compounds in the leaves, seeds, stems, and roots of the plant. Nevertheless, most of the nutraceutical properties of this plant are associated with glucosinolates and isothiocyanates [48,49].

*Moringa oleifera* contains high amounts of aromatic glucosinolates, both in the leaves and roots. The main glucosinolate is glucomoringin (a rhamnopyranosylbenzylglucosinolate), whereas moringin is a group representative of isothiocyanate. Anti-inflammatory and antioxidant activity of isothiocyanates has been reported due to their capacity to activate detoxification enzymes [50]. The polyphenol content of *Moringa oleifera* depends on the plant part. The dried leaves of *Moringa oleifera* contain many polyphenols, including flavonoids and phenolic acids that are their principal compounds [41]. Their concentrations range from 2090 to 12,200 mg GAE/100 g of DW (or 1600 to 3400 mgTAE/100 g of DW). These amounts are more significant than those found in fruit and vegetables. The observed flavonoids are mainly quercetin and kaempferol, isorhamnetin, and their glycosidic forms [42,44,51], while the phenolic acids are mainly gallic, chlorogenic, ellagic, and ferulic acids [52].

Based on the available literature, about 20 pharmacological properties can be attributed to this plant [53]. It is evident that various *Moringa* extracts can have hypoglycemic effects in different in vitro and in vivo models [48,54,55]. The antihyperglycemic effect of various aqueous-ethanol extracts of *Moringa* leaves (95, 75, 50 and 25% *v/v* and 100% water) was examined in STZ-induced diabetic rats. The most active extracts were further subjected to five liquid–liquid fractionations (hexane, chloroform, ethyl acetate, butanol, and water) and were evaluated for their antihyperglycemic activities. Among all the extracts tested, 95% (*v/v*) ethanol extract at 1000 mg/kg and butanol fraction at 500 mg/kg reduced blood glucose concentration acutely in the diabetic rats [56,57]. Bamagous et al. demonstrated that ethyl acetate extract of *Moringa* leaves at a dose of 200 mg/kg administrated to streptozotocin(STZ)-induced diabetic rats significantly decreased their blood glucose and glycosylated hemoglobin (HbA1c) levels compared to the control group [58]. Similar results were obtained in other studies using *Moringa* leaf powder at 50 mg/day. Administration of this extract to alloxan-induced diabetic rats for eight weeks triggered a significant decrease in blood glucose concentration compared with untreated diabetic rats [59].

Considering these results, the preferred doses of *Moringa* leaves used in most of these studies were 100, 200, and 300 mg/kg body weight. The long-term effect of *Moringa* leaves on glycemia in animals was evident. Additionally, no antagonistic effect of *Moringa* leaves intake was found in these long-term studies. However, long-term animal studies are still limited.

A few studies on the hypoglycemic properties of *M. oleifera* extracts in humans are available. In type 2 diabetic patients (40–58 years), consuming a standard calorie-restricted diet (1200–1800 kcal) and taking sulfonylurea medication, led to a significant reduction in postprandial blood glucose levels from 210 mg/dL to 191, 174, and 150 mg/dL, respectively after the first, second, and third month of supplementation. Moreover, in only the T2DM group, HbA1c significantly decreased from an initial value of 7.81 to 7.4 after three months of supplementation [60]. Other results exhibited a significant reduction of both fasting (28%) and postprandial (26%) blood glucose concentrations in the diabetic subjects after 40 days of *Moringa* leaf (8 g) intake, while no changes were noticed in the control group. Fombang et al. revealed that *Moringa* leaves tea at 200 and 400 mL caused an overall decrease of 17% and 19%, respectively, in the glycemia of human subjects. The explanation of the *Moringa* tea effect was that it has a strong antioxidant potential which may enhance its antihyperglycemic effect [61].

Recently, there were numerous reports on *Moringa* leaf extract as a food additive. The antidiabetic effect of *Moringa* leaf powder (20 g) incorporated into a traditional meal versus control meal was shown. After consuming the meal containing *Moringa* leaf powder, a significant decrease in blood glucose concentration at 90, 120, and 150 min in diabetic subjects compared to the control meal was detected. It had no effect on the blood glucose concentration of the healthy subjects [62].

The aforementioned human studies emphasize the antidiabetic potential of *Moringa* leaves, which were administered in different forms: capsule, tea, and food. The following doses of 7 and 8 g (as leaf powder), 200 and 400 mL (as tea), 20 g (as leaf powder incorporated into a traditional meal), and 5% *w/w* (as leaf powder incorporated into cookies) were used. However, these studies are limited in number because of different study designs and short sample sizes and durations [61,62].

The antidiabetic activity of *Moringa oleifera* may encompass various mechanisms of action, including the stimulation of insulin secretion, inhibition of α-amylase and α-glucosidase activities, decrease of gluconeogenesis in the liver, increase of glucose uptake in the muscles and liver, inhibition of glucose uptake from the intestine, and antioxidative properties. The antidiabetic activity of this plant may be the result of alleviating insulin resistance, either by neutralizing oxidative stress or by attenuating inflammation. The evidence regarding this antidiabetic plant and its phytochemicals acting directly on insulin activation signaling is very limited in the literature. The antidiabetic properties of *Moringa* leaves are attributed to the presence of small bioactive molecules: (4-hydroxyphenylacetonitrite (1), fluoropyrazine (2), methyl-4-hydroxybenzoate (3), vanillin (4), 4-α-L-rhamnopyranosylbenzylisothiocyanate (5), and 3,4-dihydroxy benzonitrile (6), as well as phenolics and flavonoids such as gallic acid (7) and rutin (8).

Hafizur et al. revealed that compounds (1)–(4) exhibited a significant glucose-dependent insulin release at a stimulatory glucose concentration of 16.7 mM, with a concomitant dose-dependent release of insulin at 200 μM. It has been suggested that possible mechanisms coupled to insulin secretion include the role of protein kinase A-mediated insulin secretion from pancreatic β-cells [63]. In the study by Attakpa et al. on diabetic mice, *Moringa* leaf extract improved insulin sensitivity by stimulating the insulin-dependent Akt pathway and upregulating glucose transporter GLUT4 expression in the muscles [64]. Furthermore, in diabetic rats consuming *Moringa* leaf extract, improved glycogen synthase activities, glycogen contents, and glucose uptake in the liver and muscles were detected [65]. 

It is believed that MO may act as an antidiabetic agent by reducing glucose levels. The underlying mechanism of this action is linked to quercetin, which can act as an inhibitor of GLUT2 [66]. No effect on GLUT5 or SGLT1 was noticed [67]. Moreover, quercetin may activate adenosine monophosphate-activated protein kinase (AMPK) to increase glucose uptake through stimulation of GLUT4 in skeletal muscle and to decrease the production of glucose through downregulation of phosphoenolpyruvate carboxykinase (PEPCK) and glucose-6-phosphatase(G6Pase) in the liver [68].

According to Waterman, after consumption of isothiocyanate-rich *Moringa* leaf extract, decreased plasma insulin, insulin resistance, and liver gluconeogenesis were detected in mice fed a very high-fat diet [69]. In different research, the intake of *Moringa* leaves caused a decrease in gastric emptying in GK rats, and this hypoglycemic effect could be linked to the presence of quercetin-3-glucoside and fibre content in the leaves [70].

In the literature, research results demonstrated the effect of *Moringa* leaf extract on the inhibition of α-amylase and α-glucosidase enzymes involved in the digestion of sugars in the intestine [71,72], however, the results regarding the effect of *Moringa* leaves on insulin secretion are inconsistent. Some authors showed the positive effect of those extracts [58,65], whereas some studies demonstrated no effect [72]. Another possible mechanism for the hypoglycemic properties of the aqueous extract from *Moringa* leaves is the decreasing expression of pyruvate carboxylase enzyme in the liver and regenerating damaged pancreatic β-cells and hepatocytes through its antioxidant activities [73].

It is worth noting that polyphenols from an MO aqueous leaf extract may inhibit protein oxidation, formation of AGEs, and protein cross-linking in glycation reactions [74]. The ability of polyphenols to scavenge free radicals derived from glycoxidation processes may explain their protection against protein glycation [75]. Phytochemicals from MO extract exhibited antioxidative properties and prevented the oxidation of lipids, thereby showing hypolipidemic and anti-atherosclerosis activity [38,76].

Sierra-Campos determined the effects of *Moringa oleifera* leaf extract on rat paraoxonase 1 (rPON1) and catalase (rCAT) activities in alloxan-induced diabetic rats. These extracts may probably activate both rPON1 and rCAT due to the influence of the specific flavonoids on the enzyme structure. The molecular blind docking analysis showed that rPON1 might have two binding sites for flavonoids. Moreover, flavonoids maybe bound at four sites in rCAT [77].

In diabetic rats treated with *M. oleifera*, a significant decrease of serum NFkβ levels and the upregulation of BCL-2 in both the kidney and liver were observed. Additionally, interleukin levels in the kidney (IL-18) and liver (IL-1α, IL-18) decreased [78].

Beyond the abovementioned activity, others have been revealed. Methanolic extract of *Moringa oleifera* leaves at adose of 200 mg/kg per body weight, inhibited by approximately 64% cell growth in Ehrlich ascites carcinoma cells in Swiss albino mice and caused upregulation of the pro-apoptotic gene Bax and tumor suppressor gene p53. It also downregulated the anti-apoptotic gene Bcl-2, thus indicating the extract’s anticancer activity [79]. Silver nanoparticles (Ag-NPs) biologically synthesized from *Moringa oleifera* leaf extract possess a significantly enhanced antibacterial activity against selected pathogenic bacterial strains, which strongly indicates that *M. oleifera* could be a potential source of Ag-NPs for successful use as an antibacterial agent in the pharmaceutical and cosmetic industries [80]. *M. oleifera* plays a pivotal role in the anti-inflammation and antioxidant processes of the kidney as revealed in conducted by Akter et al.’s [81] review of the literature, which emphasized the pharmacological and therapeutic potential of *M. oleifera*, as well as prospects of *Moringa*-based effective drug development beneficial for humans.

General characteristics of the content and properties of all three described above extracts are individually presented in Table 1.

The following analysis was aimed to systematically evaluate the literature and indicate potent plant candidates for a restricted meta-analysis. We applied Cochrane guidelines to investigate the efficacy of oral supplementation of subjected plant origin extracts in diabetes mellitus management in animal model studies. The initial literature search revealed about 300 original papers indicating three potent plant extracts for further analysis, of which only 23 articles were finally included in the restricted meta-analysis, revealing the experimentally confirmed in vivo and in vitro antidiabetic properties of *Gymnema montanum* (9 articles), *Momordica charantia* (7 articles) and *Moringa oleifera* (7 articles). In the meta-analysis protocol, data on physiological and oxidative stress parameters extracted from the original papers were examined and statistically analyzed. After any needed proper adjustments using RevMan 5.4. The categories of the analyzed parameters and observed tendencies of changes are presented in Table 2.

## 2. Meta-Analysis

### 2.1. Literature Search, Data Screening and Extraction Protocol

A comprehensive search of PubMed, MEDLINE, the Cochrane Library and Google Scholar databases was performed to classify studies reporting oral supplementation with various plant-derived extracts in rats with induced DM. Two independent investigators conducted the screening procedure, in which studies published after the year 2000 were considered suitable for analysis.

All steps of the meta-analysis were performed with high precision according to Cochrane guidelines for the systematic review of interventions [101]. The initial search procedure with quotation constructed as follow: “Plant extracts [AND] rats [AND] Diabetes mellitus” revealed 1982 papers, the screening of which led to the identification of numerous extracts that were considered potentially relevant.

Further steps consisted of a detailed screening based on the following inclusion criteria:

(1) original paper from an interventional experiment conducted on the suitable animal model (induced diabetic rats);
(2) experiment design involving the intervention group and control group either with or without a standard antidiabetic drug intervention;
(3) compatibility in the range of analyzed parameters and the manner of results presentation.

To fulfill the Cochrane criteria, two independent investigators working in parallel extracted the following characteristics from full-text papers, according to a standardized data extraction protocol: the surname of the lead author, year of publication, size of experimental/control/drug groups, study duration, study design, inclusion and exclusion criteria for each experiment measured and analyzed outcomes (primary and secondary).

**Figure 1:**
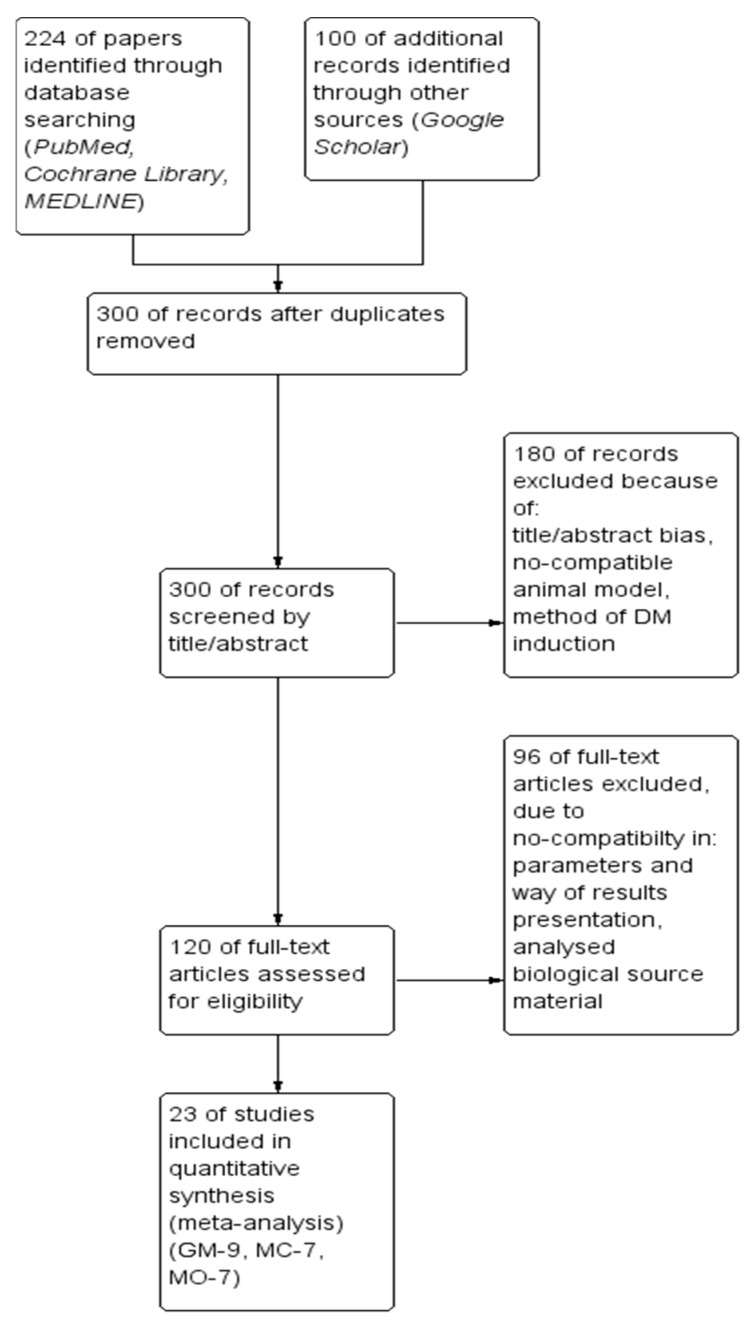
The flow diagram of the study selection procedure with numerical data about the inclusion and exclusion protocol.

After this step, the 300 original papers were determined suitable and relevant for further analysis, revealing three mentioned previously plant extracts as potent candidates for meta-analysis. Finally, only 23 met the inclusion criteria and were further meta-analyzed.The workflow of the screening procedure is presented in Figure 1.

The continuous data, after adequate transformation, were presented with the use of the same scales. The analyses were performed using the Mean Difference (MD). Furthermore, the results were presented with 95% confidential intervals (CI). No dichotomous data were analyzed. Presented values for all analyzed parameters were defined in the endpoint of the intervention termination day and presented as mean values with standard deviation (SD). In studies using more than one dose of extracts, the most effective one (as determined by authors of publications) was applied for this meta-analysis. The comparison was conducted with the random-effect model, while heterogeneity was assessed with χ^2^ and I^2^ tests. All extracted data were analyzed using the statistical software package—RevMan 5.4 (Review Manager, Copenhagen, Denmark: the Nordic Cochrane Centre). Forest plots were used to visualize the final meta-analysis results. A *p*-value < 0.05 was considered statistically significant in all analyses.

The total number of rats included in all experiments was 409 (intervention-treated: 175, control: 186, drug-treated: 48). The distribution of the number of rats in particular groups were as follows: (1) intervention groups: *G. montanum*–60, *M. charantia*–48, *M. oleifera*–67; (2) control groups: *G. montanum*–71, *M. charantia*–48, *M. oleifera*–67; (3) drug-treated groups: *G. montanum*–48.

Considering the physiological efficacy parameters, the following number of papers reported the particular selected parameters: glycemia, 20 articles (*G. montanum*–9, *M. charantia*–5, *M. oleifera*–6);insulinemia, 14 articles (*G. montanum*–9, *M. charantia*–3, *M. oleifera*–2);body weight, 10 articles (*G. montanum*–4, *M. charantia*–3, *M. oleifera*–3);food intake, 4 articles (*G. montanum*); glucose uptake by diaphragm, 3 articles (*M. oleifera*). In the case of oxidative stress, the following number of papers reported the selected parameters: TBARS, 2 articles (*G. montanum*); hydroperoxides, 2 articles (*G. montanum*); SOD, 3 articles (*M. oleifera*); and CAT, 3 articles (*M. oleifera*).

### 2.2. The Results for Physiological Parameters Analysis

**Figure 2:**
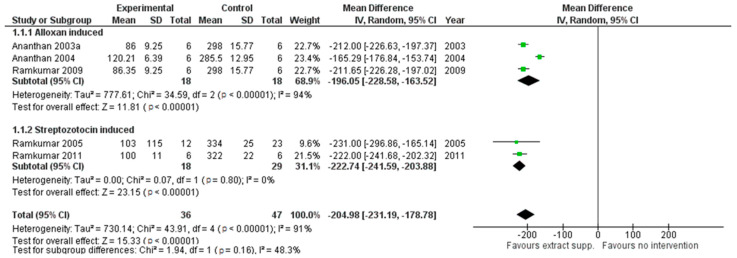
The results of *G. montanum* supplementation on glycemic levels in serum of induced diabetic rats (Forest plot).

Reduction of glycemia was observed in three cases indicating the benefits of all three extracts for supplementation in glycemic control as pooled MD, respectively, for *G. montanum*: −204.98 (95% CI [−231.18, −178.78]; *p*<0.00001) (see Figure 2); and *M. charantia*: −121.68 (95% CI [−152.50, −90.86]; *p* < 0.00001)]. The results for *M. oleifera* were inconsistent and should be interpreted with caution. Extract supplementation also revealed more robust hypoglycemic activity in comparison with the antidiabetic drug Glybenclamide in the case of *G. montanum* extracts, pooled MD: −57.71 (95% CI [−86.39, −29.03], *p* < 0.0001), indicating the effects of supplementation might be superior compared with this drug.

Insulin levels increased in serum obtained from rats supplemented with *G. montanum* and *M. charantia.* The pooled MD were, respectively, *G. montanum*: 7.99 (95% CI [0.66, 15.32], *p* = 0.03);and *M. charantia*: 2.20 (95% CI [1.08, 3.32], *p* = 0.0001), indicating the positive impact on the secretory function of the pancreas. In the case of *M. oleifera*, the results were not precise due to the differences among the subgroups. Therefore, such results might be misleading.

Glybenclamide supplementation showed a more visible insulin increase when compared with *G. montanum* in insulin increasing effect (pooled MD: −50.26 (95% CI [−108.06, 7.84], *p* = 0.09)).

Striking changes in the body weight of rats was observed in the group supplemented with *G. montanum*, indicating the probable high calorific value of the analyzed extracts and its potential influence on animal metabolism mediated by the insulin increase. The pooled MD was 63.27(95% CI [54.96, 71.57], *p* < 0.00001), and the rest of the results did not show consistent effects of supplementation.

Reduction in food intake was observed in rats supplemented with *G. montanum* (pooled MD: −53.32 (95% CI [−108.62, 1.97], *p* = 0.06)), and when compared with the Glybenclamide (pooled MD: −2.61 (95% CI [−4.46, −0,76], *p* = 0.006)).

Other results for physiological parameters show inconsistency and should be interpreted with precautions.

### 2.3. The Results of Parameters Related to Oxidative Status Analysis

**Figure 3:**
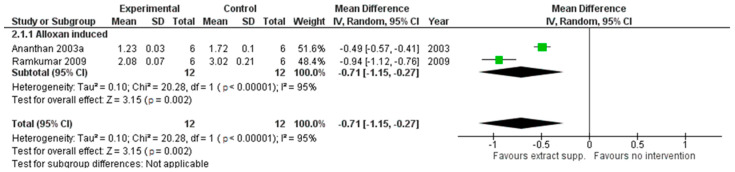
An animal model trial evaluated *G. montanum* supplementation on TBARS levels in serum of induced diabetic rats (Forest plot).

*G. montanum* extracts exhibited a lower impact on the level of TBARS (pooled MD: −0.71 (95% CI [−1.15, −0.27], *p =* 0.002)) (see Figure 3) and hydroperoxides (pooled MD: −17.33 (95% CI [−22.9, −11.75], *p* < 0.00001)) in rat plasma, revealing the positive influence on the oxidative status of animals.

In comparison with the antidiabetic drug Glybenclamide, *G. montanum* also displayed decreasing effect on hydroperoxide serum level (pooled MD: −4.95 (95% CI [−8.47, −1.44], *p* = 0.006)), while the results of the TBARS analysis were inconsistent and should be interpreted with caution. As a result of supplementation with *M. oleifera* extracts, reduced SOD activitywas observed (pooled MD: −132.23 (95% CI [−187.45, −77.00], *p* < 0.00001)), which corresponded with the reduction in oxidative stress.

Results not mentioned in the above section contained several inconsistencies, and conclusions based on them might cause incorrect outcomes.

More detailed data and results not presented above could be found in Supplementary Material (see the Supplementary Material Section).

## 3. Discussion

The therapeutic potential of plant-derived antioxidants, mostly polyphenolic compounds, is strongly evident in large studies in which plant extracts are applied to support the treatment of various diseases, including civilization, chronic diseases pertaining to obesity, diabetes mellitus or the heart [5,6,102,103,104,105,106]. Additionally, growing evidence indicates a clear link between the consumption of several plants and the prevention or treatment of chronic diseases like diabetes mellitus (DM). Thus, a healthy diet supplemented with isolated phytochemicals known for their beneficial effects can modify the risk of developing such chronic diseases [104].

Our long-term interests have focused on the molecular etiology of DM for several years and moved towards the potential application of phytochemicals with antioxidant/antidiabetic activity in diabetes treatment. Furthermore, because numerous recent studies were conducted using animal models supplemented with antioxidant/antidiabetic phytochemicals, we presented the evidence and results from a critical literature analysis followed by a strictly tailored meta-analysis of such studies.

A comprehensive search of scientific databases revealed studies reporting oral supplementation with various plant-derived extracts in rats with induced DM. The meta-analysis revealed the contribution of oral plant extract supplementation, which may trigger more detailed studies on the application of those extracts in DM management and the evaluation of the molecular basis of their action.

The final analysis was focused on antidiabetic, hypoglycemic and antioxidative activity of three plant extracts: *Gymnema montanum*, *Momordica charantia*, and *Moringa oleifera* in model rats with induced diabetes.

*G. montanum* and *M. charantia* displayed hypoglycemic, antidiabetic and antioxidative activity when supplemented to diabetic rats. Other plants belonging to the *Apocynaceae* family, such as *G. sylvestre*, and many members of the *Cucurbitaceae* family (*C. indica*, *B. hispida*, and *M. charantia* itself) are now postulated to be potent plants with antidiabetic activity [107]. Moreover, both *G. montanum* and *M. charantia* increased the insulin level, suggesting an impact on the secretory activity of β cells of the pancreas, which was confirmed by Navarrete et al. [108]. The beneficial impact of *G. montanum* and *M. charantia* on hepatic function and insulin secretion was displayed in glucose homeostasis parameters indicating their hypoglycemic activity [109].

The hypoglycemic, antihyperlipidemic and insulin increasing activity of *G. montanum* might be attributable to gymnenic acids, similar to the case of dihydroxygymnenic triacetate isolated from *G. sylvestre*, another representative of the Apocynaceae family [110].

The oxidative status of the rats treated with *G. montanum* extracts improved; therefore, the applied treatment was effective in oxidative stress (OS) reduction. *G. montanum* supplementation reduced TBARS and hydroperoxides levels significantly, resulting in a decrease in OS. Comparing *G. montanum* extract supplementation and Glybenclamide treatment gave precise results only in the case of reduced hydroperoxides produced in animals supplemented with the extract. In the case of the TBARS level, the authors did not observe any significant differences. An increase in the hexokinase activity, glucose-6-phosphate dehydrogenase and glycogen content was observed in some studies, indicating the favorable effect of *G. montanum* on glucose homeostasis [94]. As reported by Ramkumar et al., the activity of main hepatic parameters and enzymes involved in glucose metabolism indicated the positive influence of *G. montanum* on liver function.

Considering that diabetic hyperglycemia results from defects in insulin secretion and action, the above activities made extracts from *G. montanum* and *M. charantia* promising drug candidates because ofthe molecular activity of the phytochemicals included in these extracts. In summary, supplementation with the plant extracts selected for this analysis revealed their hypoglycemic activity particularly related to hepatic function improvement in glucose homeostasis, increased insulin level and the insulin sensitivity of peripheral tissues.

In some studies, animals treated with the plant extracts (particularly *G. montanum*) gained weight. It may be attributable to the high caloric value of the extract and its impact on the metabolism of rats, but it might also be interpreted as a secondary outcome of an increased insulin level, which displayed a key role in lipid metabolism. However, the induction of DM is commonly followed by weight loss in induced rats, mainly due to the catabolism of fats and proteins caused by insulin deficiency [111]. Thus, it can be postulated that the insulin-increasing activity due to supplementation with plant extracts played a decisive dual role: on the one hand, hypoglycemic; on the other hand, weight loss prevention. These aspects are of particular value when designing further trials in which a caloric value and a sugars-and-lipids pattern in the plant extracts would also be a vital parameter to consider.

In the meta-analysis, we also assessed the effects of selected plant extracts on glycemic control in animal models compared to treatment with the well-known glucose-lowering drug Glybenclamide. This oral hypoglycemic agent is a second-generation sulfonylurea derivative that increases insulin secretion, probably by interacting with sulfonylurea receptors on beta cells or by interfering with ATP-sensitive potassium channels on pancreatic beta cells. The comparison of the activity of the extracts with Glybenclamide demonstrated the favorable influence of the *G. montanum* extracts on the glucose level, precisely its superior hypoglycemic activity. Additional studies are needed to reveal the molecular mechanisms underlying the hypoglycemic activity of *G. montanum* that are different from those already described above.

Additionally, in rats supplemented with *G. montanum*, a reduction in food intake was observed compared to those treated with an oral drug. This fact might be probably related to the high caloric value and the high dietary fibre content of extracts. In the case of *Gymnema montanum*, the literature data are limited; therefore, more studies on the bioactivity of this plant, as well as its composition, are needed [92].

The analysis of the activity of antioxidant enzymes (CAT, SOD) provided additional information about the impact of supplementation on the oxidative status in rats. The *M. oleifera* extracts reduced the activity of SOD in the treated rats, lowering the oxidative stress by decreasing free-radical concentration. Some discrepancies were observed in the analysis of the CAT activity and should be interpreted with caution. However, the observed increase in CAT activity agreed with the previously reported data [99]. The *M. oleifera* extracts are rich in phytochemicals (polyphenols, vitamins, flavonoids, tannins, saponins and alkaloids) that might have been responsible for OS reduction [36,111].

Therefore, it may be assumed that the plant extracts from *Gymnema montanum*, *Momordica charantia*, and *Moringa oleifera* supplemented to rats that had induced diabetes revealed a positive influence on the oxidative status and are very potent candidates for diabetic management, accordingly to results obtained from analyzed cases.

The analysis was based on a search of the recent literature (after the year 2000) from medical databases, providing much reliable data. Two independent researchers carefully evaluated and conducted the review protocol, data extraction, and further citation, and all of those were processed according to the Cochrane guidelines [101]. The results were reported in accordance with the Preferred Reporting Items for Systematic reviews and Meta-Analyses (PRISMA) statement [111]. The analyzed parameters are vital factors reflecting the changes in physiology and function of DM-induced rats and reflect the OS status of subjects. Since our analysis was focused on critical physiological and biochemical parameters reflecting the outcomes of the intervention, the results provide precise data for drawing conclusions based on them.

Comparing different studies on plant extracts’ medical/supplementary potential is often hampered or impossible since there are significant distinctions between studies. These involve the final content of phytochemicals resulting from different extraction procedures (solvents, conditions, storage and analysis), and different criteria of diabetes recognition and experimental protocols, which display numerous variances, including study groups and controls, intervention duration, administered doses, analyzed parameters or other factors. Finally, there are many imprecisions in how results are reported. Therefore, the restricted and detailed protocol for meta-analysis allowed drawing conclusions based upon numerous independent studies. That is what we did to provide relevant and reliable results that constituted the basis for the final analysis and why we only took 23 out of 300 primarily selected papers.

Several points of criticism should be remembered when evaluating the health effects of supplementation with phytochemicals like polyphenols, which, as far as has been established, consist of a group of representative compounds (approximately 10,000) having broad diversity in structure and physiological role.

It is challenging to conclude their short- and long-term health effects from experimental and interventional data. It also has to be remembered that the biological activity of all phytochemicals is robustly dependable on many other factors such as their bioavailability in organisms and the degree of the chemical treatment during the extraction protocol, which can alter the former and further activity in the organism. The role of the recipient’s microbiota in the metabolism of phytochemicals is to alterthe activity of supplemented compounds by producing different metabotypes, which can display different activities and affect physiological functions in an alternating manner. All of the above factors cause high inter-individual differences in the biological response to supplementation. Awareness of the fact that the analyzed effect of supplementation of animals with phytochemicals was the result of multiple synergistic/antagonistic interactions of multiple dietary components, environmental factors and individual properties of subjects is a crucial factor that needs to be considered when drawing conclusions from such data. Additionally, the possible risk of the low adherence of the experimental protocol to real-life conditions, in which polyphenol consumption might be completely different, can complicate the analysis. Moreover, the restricted selection of data from trials with plant extracts conducted in chemical laboratories raises considerations about the health benefits emerging from a diet naturally enriched by polyphenols and other phytochemicals and the different synergistic effects of various components might be observed.

Despite these limitations, the results of this meta-analysis strongly encourage further studies to evaluate the impact of plant-derived extracts on diabetic patients’ physiological and oxidative status parameters. Further study to investigate the activity of these analyzed extracts in humans is strongly recommended since there is no direct way to extrapolate results obtained from animals to humans due to their complexity. There is always a risk of severe adverse events and toxicity from such supplementation in humans. It is crucial to indicate the differences between the animal model of diabetes and the physiology of human patients with this disease, and this constitutes a critical limitation at the entry point of study design and on further data analysis. The abovementioned results are promising and may trigger the further development of experimental and clinical approaches to investigate the application of these plant extracts in more advanced and detailed studies.

## 4. Conclusions

The presented meta-analysis confirmed that extracts of *Gymnema montanum*, *Momordica charantia* and *Moringa oleifera* represent a promising and attractive source of phytochemicals with proven antidiabetic and antioxidant activity in rat models of diabetes. They increase pancreatic insulin and insulin sensitivity in peripheral tissues, reduce insulin resistance and hepatic gluconeogenesis, and have a modulatory effect on glycolysis, gluconeogenesis and antihyperlipidemic properties. All three extracts reduced oxidative stress and revealed antiperoxidative features to protect β-cells against ROS. They are, therefore, good candidates for the management and treatment of diabetes in mammals, especially humans. Moreover, all three plants have been widely used in traditional medicine.

This study revealed that the application of stringent inclusion/exclusion criteria (23 out of 300 papers) displayed a lack of generally accepted standards in a description of experiments with plant extracts (a type of extraction, analysis of extract, the concentration of different phytochemicals) and experimental protocols, making a direct comparison and analysis more demanding. However, considering all the strengths and limitations, this meta-analysis is a reliable source of data and might constitute an inducement for further physiological and mechanistic studies.

This review would shed light on how plant-based drugs could potentially be a beneficial agents in treating of aging, oxidative stress and hyperglycemia-associated abnormalities.

## Figures and Tables

**Table 1:** *M. charantia*, *M. oleifera* and *G. montanum* and their characteristics.

Plant	Family	Occurrence Area	Identified Phytochemicals	Biological Activity of Extracts
*Momordica charantia*	*Cucurbitaceae* family	India, China, East Africa, Central and South America	triterpenoids and saponins (even up to 0.0432%) [82,83],	increase pancreatic insulin decrease insulin resistance [84], inhibit glucosidase, suppress the activity of disaccharides, reduce adiposity reduce xenobiotic metabolism [85] reduce oxidative stress [86] increase cytochrome P45
			polypeptides [87],	
			flavonoids and phenolics (1.77 ± 0.72%) [83],	
			alkaloids and sterols [24],	
			unsaturated fatty acids (20.1%–64.3%),	
			alkaloids, amino acids (up to 11.99%)	
			Vitamins,	
			polypeptide *charantin* [88],	
			polysaccharides (5.91% to 10.62%) [89]	
*Gymnema* *montanum*	*Apocynaceae* family	India—Western Ghats	11.57% *w/w* of carvacrol,	modulatory effect on glycolysis and gluconeogenesis [8] anti-hyperlipidemic properties [90,91] antiperoxidative properties [92,93] protect ß-cells against ROS [94,95]
			6.77% of erythritol,	
			4.58% of gallic acid,	
			and 3.09% of quercetin [19]	
*Moringa* *oleifera*	*Moringaceae* family	Africa, India	vitamins, carotenoids, polyphenols, phenolic acids, flavonoids, alkaloids, glycosylates, isothiocyanates, tannins and saponins [96,97] lipids (stearic acid, palmitic acid and oleic acid), calcium, potassium, sodium, iron [98]	reduce insulin resistance [59], reduce hepatic gluconeogenesis [99], influence β-cell mass and function, increase insulin sensitivity in peripheral tissues [100]

**Table 2:** Distribution and changes of analyzed parameters in meta-analysis.

Plant	Physiological Efficacy Parameters		Oxidative Stress Parameters	
*Momordica* *charantia*	vs. control		no data analyzed Ø	
	Glycemia **↓** Insulinemia ↑ body weight ↔ glucose uptake by diaphragm↑			
*Gymnema* *montanum*	vs. control	vs. drug	vs. control	vs. drug
	Glycemia ↓ Insulinemia ↑ body weight ↑ food intake ↓	Glycemia ↓ Insulinemia ↓ body weight ↔ food intake ↓	TBARS ↓ Hydroperoxides ↓	TBARS ↓ Hydroperoxides ↓
*Moringa* *oleifera*	vs. control		vs. control	
	Glycemia ↓ Insulinemia ↔ body weight ↑		SOD ↓ CAT ↑	

Changes of parameters in experimental group: **↓**—decrease, ↑—increase, ↔—unchanged, Ø—not analyzed.

## Supplementary Materials

The following are available online at https://www.mdpi.com/article/10.3390/cimb44020049/s1.
