# Supplementary material for: Evidence from a Systematic Review and Meta-Analysis Pointing to the Antidiabetic Effect of Polyphenol-Rich Plant Extracts from Gymnema montanum, Momordica charantia and Moringa oleifera
Source: Curr Issues Mol Biol. 2022 Jan 28;44(2):699–717. doi: 10.3390/cimb44020049 (PMC8928996; doi:10.3390/cimb44020049)
Supplement: Supplementary file 1 [file cimb-44-00049-s001.zip › cimb-1574100-supplementary.pdf]

Supplementary Material

Title: Evidence from a Systematic Review and Meta-Analysis Pointing to the Antidiabetic Effect of the Polyphenol-Rich Plant Extracts from *Gymnema montanum, Momordica charantia and Moringa oleifera*.

Authors: Michal Krawczyk[1], Izabela Burzynska-Pedziwiatr1, Lucyna Alicja Wozniak[1], Malgorzata Bukowiecka-Matusiak[1]*

[1] Chair of Medical Biology, Laboratory of Metabolomic Studies, Department of Structural Biology, Faculty of Medicine, Faculty of Biomedical Sciences, Medical University of Lodz, Zeligowskiego 7/9, 90-752 Lodz, Poland;michal.s.krawczyk@gmail.com; izabela.burzynska-pedziwiatr@umed.lodz.pl; lucyna.wozniak@umed.lodz.pl;

* Correspondence: malgorzata.bukowiecka-matusiak@umed.lodz.pl; Tel.: +48 422725240

Table of content

1. Flow diagram of study selection procedure, numerical data about the inclusion and exclusion protocol. 2

2. Detailed characteristics of all included studies. 3

3. Distribution of the analyzed parameters in meta-analysis. 6

4. Detailed results of meta-analysis of physiological parameters: 7

   4.1. *Gymnema montanum* analysis results. 7

   4.2. *Gymnema montanum* vs. Glibenclamide analysis results. 8

   4.3. *Momordica charantia* analysis results. 9

   4.4. *Moringa oleifera* analysis results. 10

5. Detailed results of meta-analysis of Oxidative status parameters: 11

   5.1. *Gymnema montanum* analysis results. 11

   5.2. *Gymnema montanum* vs. Glibenclamide analysis results. 11

   5.3. *Moringa oleifera* analysis results. 12

1. **Flow diagram of study selection procedure, numerical data about the inclusion and exclusion protocol.**

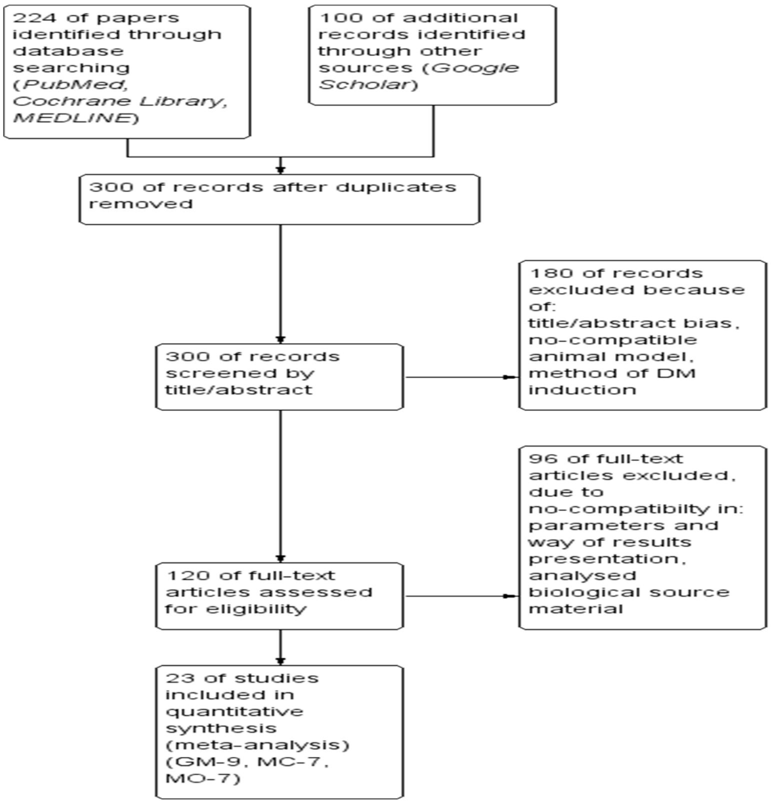

Figure S1. Flow diagram of study selection procedure, numerical data about the inclusion and exclusion protocol.

2. Detailed characteristics of all included studies.

Study identifier [references]	Diabetes induction	Animals model	Outcomes	Notes
**Gymnema montanum (GM)**				
Ananthan 2003	i.p. injection with freshly prepared solution of alloxan monohydrate in normal saline at a dose of 150 mg kg−1 BW.	36 Male albino Wistar rats 170–200 g BW	Glycaemia, Insulinaemia, T BARS and hydroperoxides in plasma, GSH, Vitamins C and E	Rats with that exhibited glycosuria and hyperglycemia (i.e. glycaemia concentration 200–300 mg dl[1]) were taken for the experiment.
Ananthan 2003a	.p. injection with freshly prepared solution of alloxan monohydrate in normal saline at a dose of 150 mg/kg BW.	42 Male albino Wistar rats, body weight of 180 to 200 g.	glycaemia, serum and tissue lipids, hexokinase, glucose-6-phosphatase, TBARS, hydroperoxides, and glutathione	As above.
Ananthan 2004	.p. injection with a freshly prepared solution of alloxan monohydrate in normal saline at a dose of 150 mg/kg of BW	54 Male albino Wistar rats, with BW of 180 to 200 g	glycaemia, insulinaemia, lipid peroxidation, reduced glutathione content and activities of CAT, SOD, GPx, and GST.	As above.
Ramkumar 2005	i.p. injection with STZ in citrate buffer (pH =4.5) at a dose of 100 mg/kg BW.	30 Male albino Wistar rats (170 to 200g each), aged 48 ± 2 h,	glycaemia, insulinaemia, lipid peroxidation, reduced glutathione content and activities of CAT, SOD, GPx, and GST.	After 12 wks, male rats weighing above 150 g were selected for screening in the NIDDM model.
Ramkumar 2007	i.p. injection with a freshly prepared solution of alloxan monohydrate in normal saline at a dose of 150 mg/kg of BW	Male albino Wistar rats (BW, 170-200 g)	glycaemia, insulinaemia, OGTT and lasma/tissue glycoproteins, hexosamine, sialic acid, and fucose	As above.
Ramkumar 2008	i.p. injection with freshly prepared solution of alloxan monohydrate in normal saline at a dose of 150 mg/kg BW	42 Male albino Wistar rats of 170–200 g BW	Glycaemia, OGTT, insulinaemia, TBARS, MDA, and Hydroperoxides,SOD, GPx, and CAT activities	As above.
Ramkumar 2008a	i.p. injection with freshly prepared solution of alloxan monohydrate in normal saline at a dose of 150 mg/kg BW	Male adult albino Wistar rats, 12 wks old, BW 180–	lipid profile, lipoprotein changes and fatty acid composition, TC,TG, FFA, The ratio between HMG-CoA and mevalonate in hepatic tissue as an index of the activity of HMG-CoA reductase	As above.
Ramkumar 2009	Diabetes was induced in the rats and administering alloxan.	30 Male albino Wistar rats of 170–200 g BWs	glycaemia, insulinaemia, renal markers including urea, creatinine and uric acid, lipid peroxidation markers including TBARS and hydroperoxides and antioxidant enzymes SOD, CAT, GPx and GST activities in kidney	As above.
Ramkumar 2011	Single i.p. injection of STZ (60 mg/kg bw). Control animals received only citrate buffer	48 Male albino Wistar rats (170–200 g BW)	glycaemia levels and insulinaemia, activity of hexokinase, Glucose-6-phosphate dehydrogenase and glycogen content in liver, levels of glucose-6-phosphatase and fructose-1,6-bisphosphatase.	injection, animals with fasting glycaemia above 250 mg/dl were considered as diabetic and included in the

Study identifier [references]	Diabetes induction	Animals model	Outcomes	Notes
**Momordica charantia (MC)**				
Atila 2015	i.p. injection with STZ	40 Sprague Dawley rats, aged 5-6months	Glycaemia TOS and TAS levels in plasma and erythrocytes, LDL, HDL, VLDL, TC, TG	----
El Batran 2006	i.p. injection of 150 mg/kg of a 5% aqueous solution of alloxan	adult albino rats of both sexes " Sprague–Dawley" weighting 120–150 g	Serum glucose, creatinine, urea, Serum alkaline Phosphatase, transaminases " AST and ALT" Serum TG and TC	----
Fernandes 2007	s.c., injection of alloxan monohydrate (100 mg/kg) in acetate buffer (pH 4.5).	32 Albino rats of the Wistar strain, of either sex, weighing 150–200 g,	Glucose tolerance test, glycosylated haemoglobin, mean glycaemia, serum insulin, TC,TG, protein and glycogen content of liver. The hemidiaphragms and livers glucose uptake/transfer processes, Histopathological study of pancreas.	----
Kar 2003	i.p. injection of alloxan monohydrate to overnight fasted animals at a dose of 100 mg/kg BW by partially destroying pancreatic beta cells.	160 Charles Foster strain male albino rats (BW. 150- 200 g) were used throughout the studies.	Glycaemia, Urine sugar	Steady diabetes was confirmed noting urine sugar regularly and then measuring glycaemia values before starting an experiment.
Mahmoud 2017	i.p. injection of a single dose of freshly prepared STZ (45 mg/kg) in citrate buffer (0.09 M, pH 4.8)	Adult male albino rats (aged 6–8 wks) weighing 150–200 g	glucose uptake of isolated rat diaphragm muscles in the presence and absence of insulin. Histopathological examination of pancreas; Serum glucose level, TC,TG and HDL-cholesterol levels, Serum insulin level, Serum fructosamine level, TAOC, Pancreatic MDA	rats with persistent glycaemia levels 200 mg/dL, for 7 days after STZ administration, were considered diabetic and included in the study
Mhammady 2012	i.p. injection of a single dose of 100 mg/kg BW alloxan monohydrate dissolved in citrate buffer at pH 4.5.	50 adult male albino rats weighing about 120-160g	OGTT, serum insulin, lipid profiles, HbA1c%, liver enzymes activity and glycogen content, intestinal absorption and diaphragm uptake of glucose and histopathological studies on the pancreas were evaluated, serum ALT and AST activity, and lipid profiles.	Rats having serum glucose ranging from 180-300 mg/dl after 2 hours of glucose intake were only included in the experiment.
Poonam 2013	i.p.injection with streptozotocin. (50 mg kg^{-1}) freshly prepared in 0.1M sodium citrate buffer.	30 Healthy adult rats of wistar strain were used in the present study	Glycaemia, BW	The diabetic state was confirmed 48 h after STZ injection. Threshold value of fasting glycaemia was taken as >200 mg dL^{-1}

Study identifier [references]	Diabetes induction	Animals model	Outcomes	Notes
***Moringa oleifera* (MO)**				
Al-Maki 2015	i.v. injection of STZ(60mg/kg in 0.1mol/l citrate buffer), control group consisted of 10 rats injected with 0.1mol/L citrate buffer	40 adult male Albino rats weighing 180-200g	Glycaemia level, BW; SOD and CAT level in kidney tissue homogenate	Rats with glycaemia higher than 200mg/dL after the 5 days from injection was considered as diabetic.
Gupta 2012	i.p. injection with STZ (50mg/kg BW), control rats (3 groups of 7 animals) were injected with 0.1mmol/L sodium citrate buffer alone	9 Colony bred, 28 sexually mature albino Wistar rats weighing 170-230g,	Glycaemia level, insulinaemia level	Rats were considered as diabetic when fasting glucose were >250mg/dl..
Jaiswal 2013	Single i.p. injection of freshly prepared STZ at a dose of 55 mg/kg BW	30 Male albino Wistar rats weighing 180-220 g	SOD, CAT, GST, LPO,	Rats with marked hyperglycemia in terms of fasting blood glucose (FBG) and postprandial glucose (PPG) [FBG>250mg/dL and PPG> 350mg/dL were used in the study].
Kar 2003	i.p. injection of alloxan monohydrate to overnight fasted animals at a dose of 100 mg/kg BW by partially destroying pancreatic beta cells.	160 Charles Foster strain male albino rats (wt. 150- 200 g) were used throughout the studies.	Glycaemia, Urine sugar	Steady diabetes was confirmed noting urine sugar regularly and then measuring glycaemia values before starting an experiment
Olurishe 2016	single i.p. injection with alloxan, dose of 150mgkg BW administered i.p.	56 Wistar rats of both sexes	Glycaemic control parameters, insulin level, BWs, lenticular morphology	Rats with the glycaemia level greater than 150mg/dL were considered diabetic, rats with glycaemia over 250mg/dL were considered as post-prandial hyperglycaemic
Omabe 2014	i.p. injection with alloxan Monohydrate dissolved in sterile PBS of dose 84mg/kg	Adults albino rats of both sexes and the same age group (8–12 wks) weighing 130–200 g	Glycaemia Level, Electrolytes Determinatio,Plasma Bicarbonate, Lactate Dehydrogenase Level,	only animals with fasting blood glucose 11–20mmol/L were considered diabetic used for the experiment.
Omodanisi 2017	i.p. injection of single dose of streptozotocin (55 mg/kg)	forty-eight (48) adult male Wistar strain	MDA levels, CAT, GPx, SOD activities, GSH and inflammatory biomarkers TNF-α, IL-6) were determined in the kidney.	----

3. Distribution of the analyzed parameters in meta-analysis.

Table S1. Distribution of analysed parameters in meta-analysis.

<table>
<tr><th rowspan="2">Plant</th><th colspan="2">Physiological efficacy parameters</th><th colspan="2">Oxidative stress parameters</th></tr>
<tr><th>vs control</th><th>vs drug</th><th>vs control</th><th>vs drug</th></tr>
<tr><td>Gymnema montanum</td><td>glycemia ↓
insulinemia ↑
body weight ↑
food intake↓</td><td>glycemia↓
insulinemia↓
body weight ↔
food intake ↓</td><td>TBARS ↓
hydroperoxides ↓</td><td>TBARS ↓
hydroperoxides ↓</td></tr>
<tr><td>Momordica charantia</td><td colspan="2">vs. control

glycemia ↓
insulinemia ↑
body weight ↔
glucose uptake by diaphragm ↑</td><td colspan="2">No parameters analyzed ∅</td></tr>
<tr><td>Moringa oleifera</td><td colspan="2">vs. control

glycemia ↓
insulinemia ↔
body weight ↑</td><td colspan="2">vs. control

SOD ↓
CAT ↑</td></tr>
</table>

5. Detailed results of meta-analysis of physiological parameters

5.1. *Gymnema montanum* analysis results.

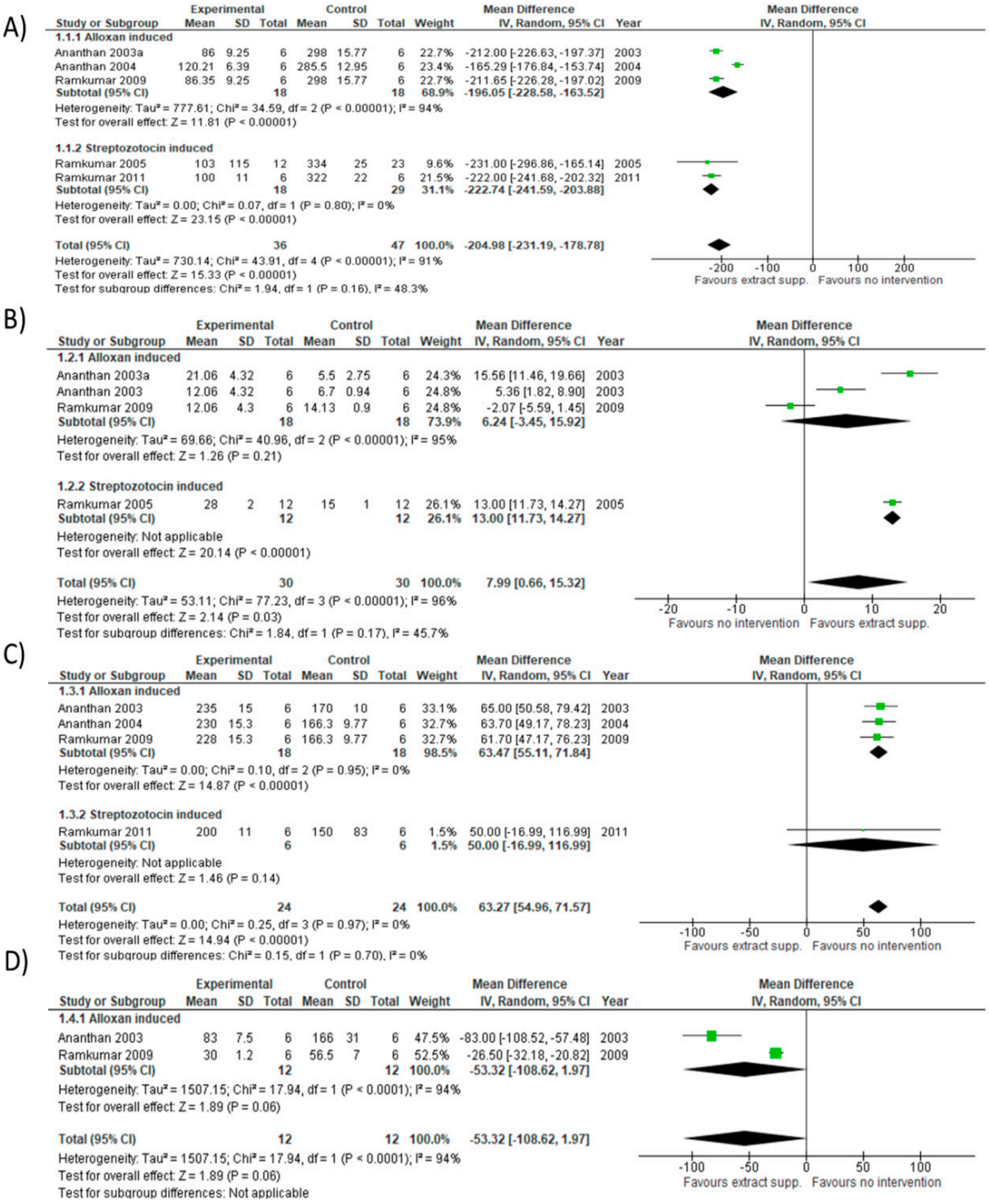

Figure S2. Results of meta-analysis of physiological parameters for *Gymnema montanum* extract: A) Glycemia, B) Insulinemia, C) Change in Body weight, D) Food intake.

5.2. *Gymnema montanu*m vs. *Glibenclamide* analysis results.

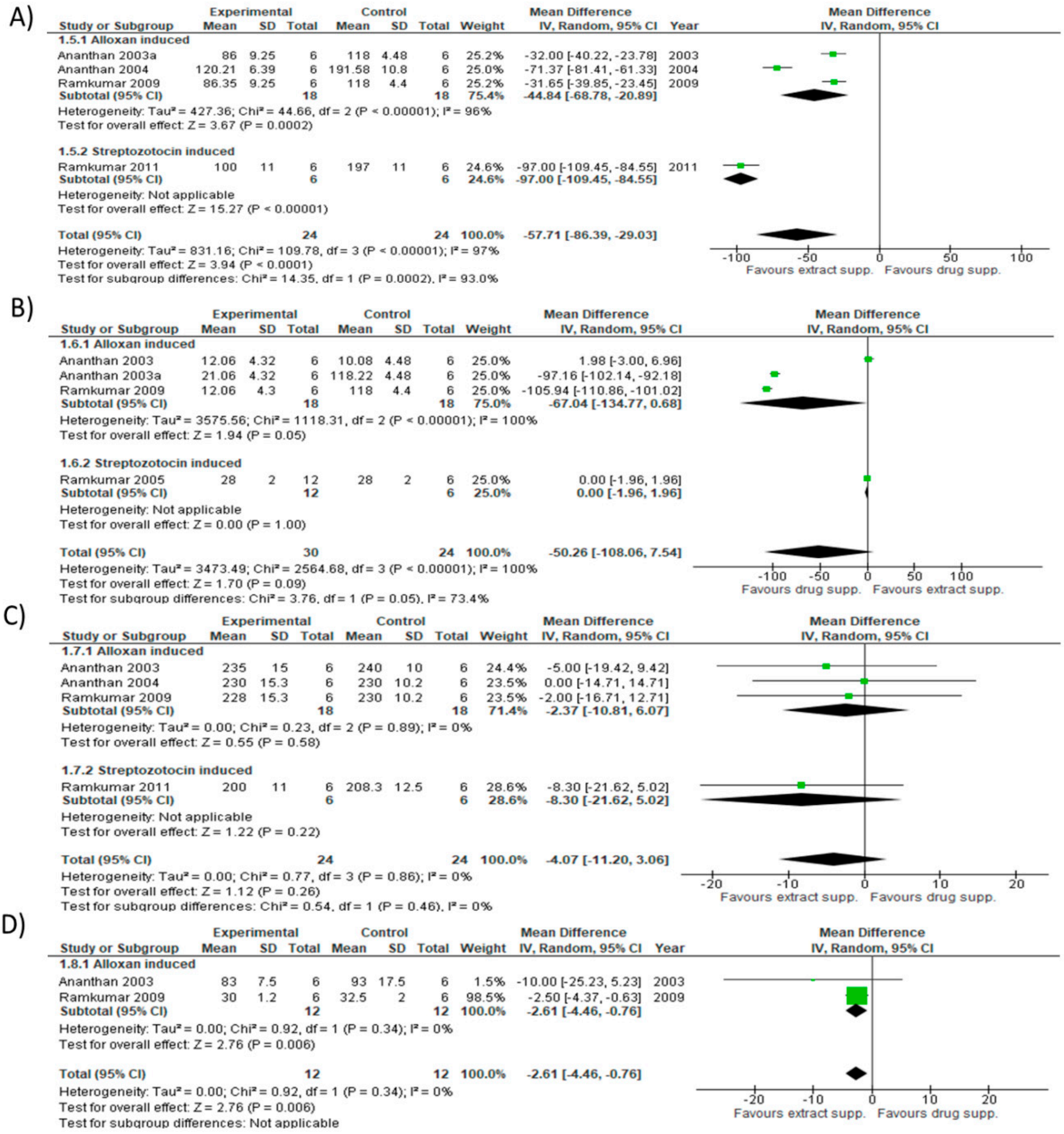

Figure S3. Results of meta-analysis of physiological parameters for *Gymnema montanum* extract comparison with Glybenclamide: A) Glycemia, B) Insulinemia, C) Change in Body weight, D) Food intake.

5.3. *Momordica charantia* analysis results.

A)

Study or Subgroup	Experimental Mean	Experimental SD	Experimental Total	Control Mean	Control SD	Control Total	Weight	Mean Difference IV, Random, 95% CI
**1.2.1 Alloxan induced**								
El Batran 2006	149.3	10.4	6	235.8	10.8	6	28.1%	-86.50 [-98.50, -74.50]
Fernandes 2007	152.4	33	8	295.5	33	8	22.2%	-143.10 [-175.44, -110.76]
Kar 2003	119	9	5	250	22	5	25.9%	-131.00 [-151.83, -110.17]
**Subtotal (95% CI)**			**19**			**19**	**76.3%**	**-118.34 [-155.83, -80.84]**
Heterogeneity: Tau² = 965.44; Chi² = 20.06, df = 2 (P < 0.0001); I² = 90%								
Test for overall effect: Z = 6.19 (P < 0.00001)								
**1.2.2 Streptozotocin induced**								
Atila 2015	263.83	22.82	10	397	38.56	10	23.7%	-133.17 [-160.94, -105.40]
**Subtotal (95% CI)**			**10**			**10**	**23.7%**	**-133.17 [-160.94, -105.40]**
Heterogeneity: Not applicable								
Test for overall effect: Z = 9.40 (P < 0.00001)								
**Total (95% CI)**			**29**			**29**	**100.0%**	**-121.68 [-152.50, -90.86]**
Heterogeneity: Tau² = 841.15; Chi² = 24.40, df = 3 (P < 0.0001); I² = 88%								
Test for overall effect: Z = 7.74 (P < 0.00001)								
Test for subgroup differences: Chi² = 0.39, df = 1 (P = 0.53), I² = 0%								

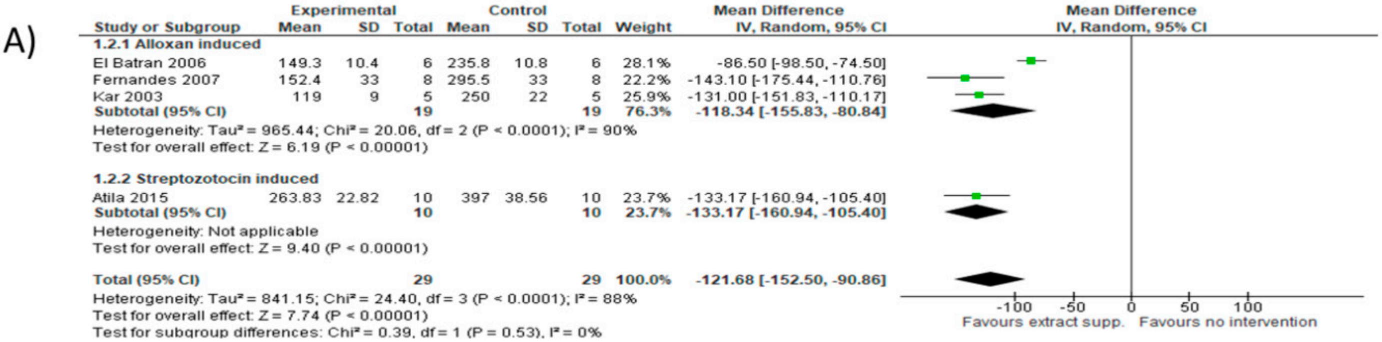

B)

Study or Subgroup	Experimental Mean	Experimental SD	Experimental Total	Control Mean	Control SD	Control Total	Weight	Mean Difference IV, Random, 95% CI
**1.1.1 Alloxan induced**								
Fernandes 2007	2.6	0.3	6	1.6	0.2	6	37.9%	1.00 [0.71, 1.29]
Mohammady 2012	12.5	2.12	10	6.3	0.87	10	23.9%	6.20 [4.78, 7.62]
**Subtotal (95% CI)**			**16**			**16**	**61.7%**	**3.55 [-1.54, 8.65]**
Heterogeneity: Tau² = 13.25; Chi² = 49.45, df = 1 (P < 0.00001); I² = 98%								
Test for overall effect: Z = 1.37 (P = 0.17)								
**1.1.2 Streptozotocin induced**								
Mahmoud 2017	3.28	0.08	6	2.39	0.27	6	38.3%	0.89 [0.66, 1.12]
**Subtotal (95% CI)**			**6**			**6**	**38.3%**	**0.89 [0.66, 1.12]**
Heterogeneity: Not applicable								
Test for overall effect: Z = 7.74 (P < 0.00001)								
**Total (95% CI)**			**22**			**22**	**100.0%**	**2.20 [1.08, 3.32]**
Heterogeneity: Tau² = 0.84; Chi² = 52.39, df = 2 (P < 0.00001); I² = 96%								
Test for overall effect: Z = 3.86 (P = 0.0001)								
Test for subgroup differences: Chi² = 1.05, df = 1 (P = 0.31), I² = 4.4%								

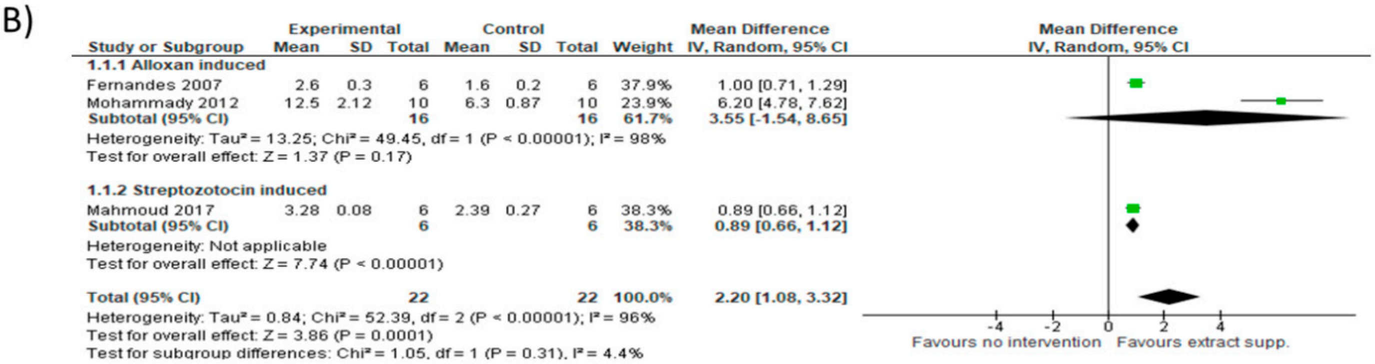

C)

Study or Subgroup	Experimental Mean	Experimental SD	Experimental Total	Control Mean	Control SD	Control Total	Weight	Mean Difference IV, Random, 95% CI
**1.3.1 Alloxan induced**								
El Batran 2006	-2.37	28.5	6	19.23	59.47	6	27.7%	-21.60 [-74.37, 31.17]
Mohammady 2012	6.4	2.22	10	-12.3	2.81	10	72.3%	18.70 [16.48, 20.92]
**Subtotal (95% CI)**			**16**			**16**	**100.0%**	**7.53 [-27.83, 42.88]**
Heterogeneity: Tau² = 448.99; Chi² = 2.24, df = 1 (P = 0.13); I² = 55%								
Test for overall effect: Z = 0.42 (P = 0.68)								
**Total (95% CI)**			**16**			**16**	**100.0%**	**7.53 [-27.83, 42.88]**
Heterogeneity: Tau² = 448.99; Chi² = 2.24, df = 1 (P = 0.13); I² = 55%								
Test for overall effect: Z = 0.42 (P = 0.68)								
Test for subgroup differences: Not applicable								

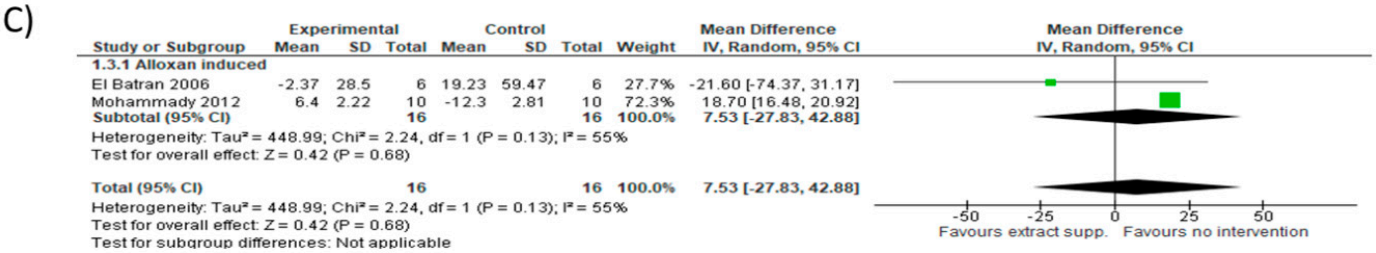

D)

Study or Subgroup	Experimental Mean	Experimental SD	Experimental Total	Control Mean	Control SD	Control Total	Weight	Mean Difference IV, Random, 95% CI
**1.4.1 Alloxan induced**								
Fernandes 2007	15.9	0.4	8	4.7	0.2	8	33.4%	11.20 [10.89, 11.51]
Mohammady 2012	17.2	1.52	10	12	1.38	10	33.1%	5.20 [3.93, 6.47]
**Subtotal (95% CI)**			**18**			**18**	**66.6%**	**8.23 [2.35, 14.11]**
Heterogeneity: Tau² = 17.78; Chi² = 80.63, df = 1 (P < 0.00001); I² = 99%								
Test for overall effect: Z = 2.74 (P = 0.006)								
**1.4.2 Streptozotocin induced**								
Mahmoud 2017	3.94	0.26	6	2.54	0.47	6	33.4%	1.40 [0.97, 1.83]
**Subtotal (95% CI)**			**6**			**6**	**33.4%**	**1.40 [0.97, 1.83]**
Heterogeneity: Not applicable								
Test for overall effect: Z = 6.38 (P < 0.00001)								
**Total (95% CI)**			**24**			**24**	**100.0%**	**5.94 [-1.50, 13.37]**
Heterogeneity: Tau² = 42.97; Chi² = 1330.12, df = 2 (P < 0.00001); I² = 100%								
Test for overall effect: Z = 1.57 (P = 0.12)								
Test for subgroup differences: Chi² = 5.16, df = 1 (P = 0.02), I² = 80.6%								

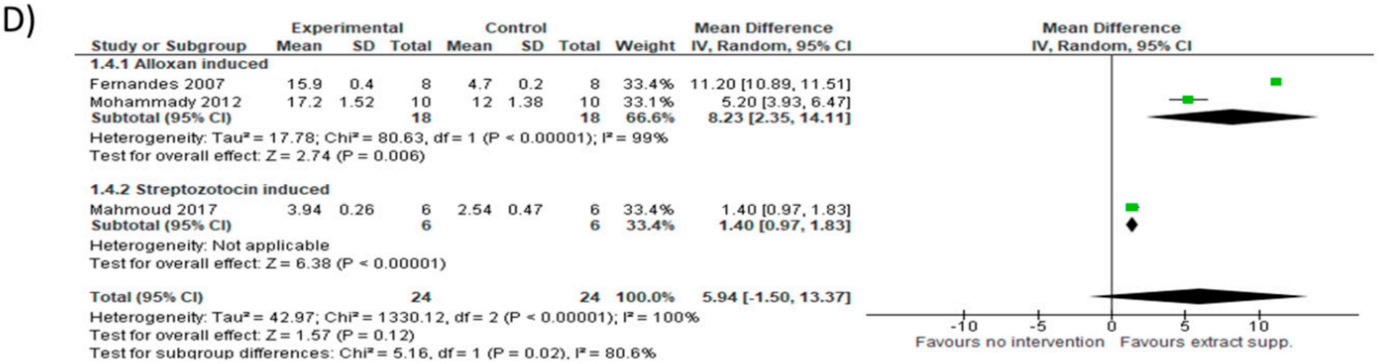

Figure S4. Results of meta-analysis of physiological parameters for *Momordica charantia* extracts: A) Changes in Glycemia, B) Changes in Insulinemia, C) Changes in Body weight, D) Glucose uptake by the diaphragm.

5.4. *Moringa oleifera* analysis results.

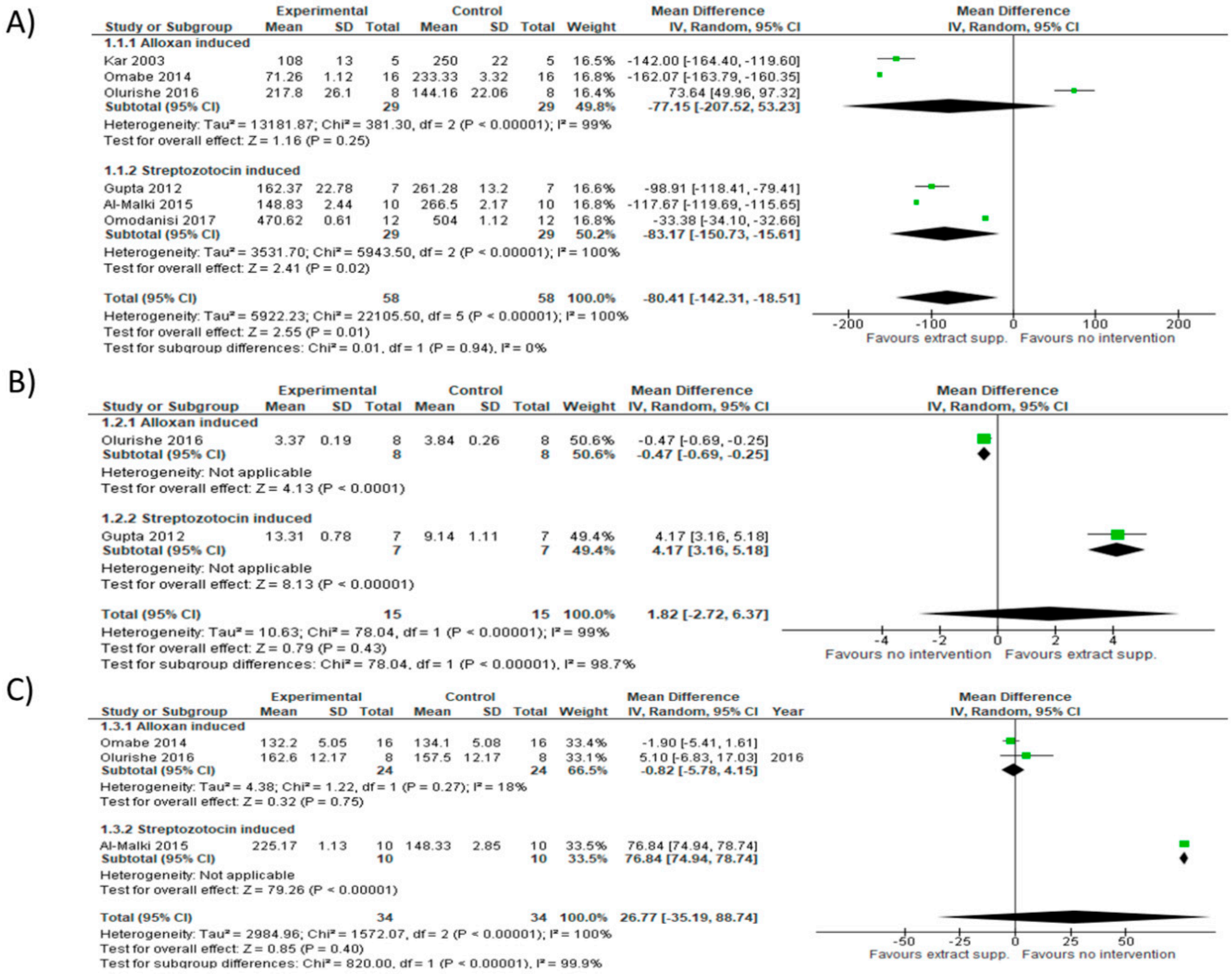

Figure S5. Results of meta-analysis of physiological parameters for *Moringa oleifera* extracts:
A) Changes in Glycemia, B) Changes in Insulinemia, C) Changes in Body weight.

6. Detailed results of meta-analysis of Oxidative status parameters:

6.1. Gymnema montanum analysis results.

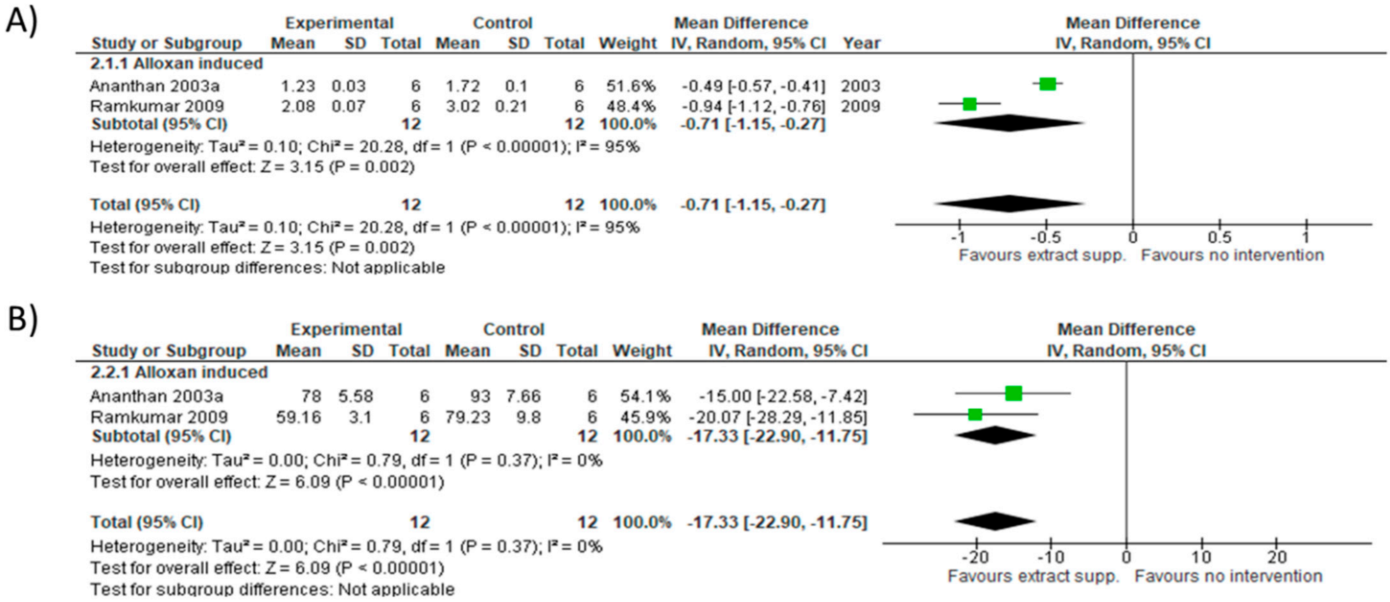

Figure S6. Results of meta-analysis of oxidative status parameters for *Gymnema monta*num extracts: A) Change in TBARS level, B) Change in Hydroperoxides level.

6.2. *Gymnema montanum* vs. Glibenclamide analysis results.

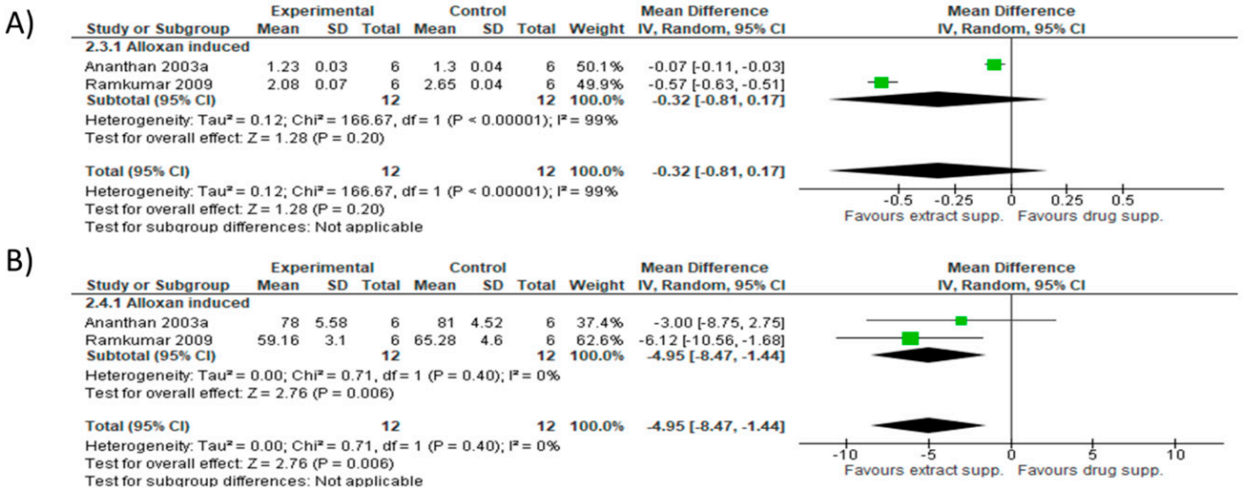

Figure S7. Results of meta-analysis of oxidative status parameters for *Gymnema mont*anum extracts in comparison with Glibenclamide: A) Changes in TBARS level, B) Changes in Hydroperoxides level.

6.4. *Moringa oleifera* analysis results.

A)

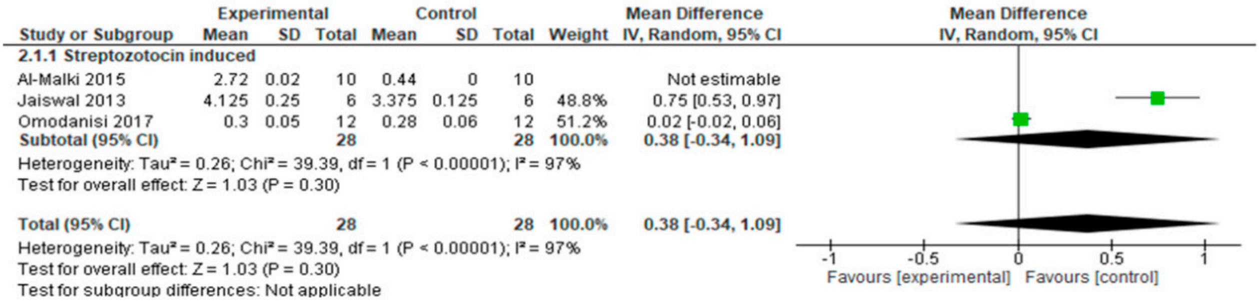

B)

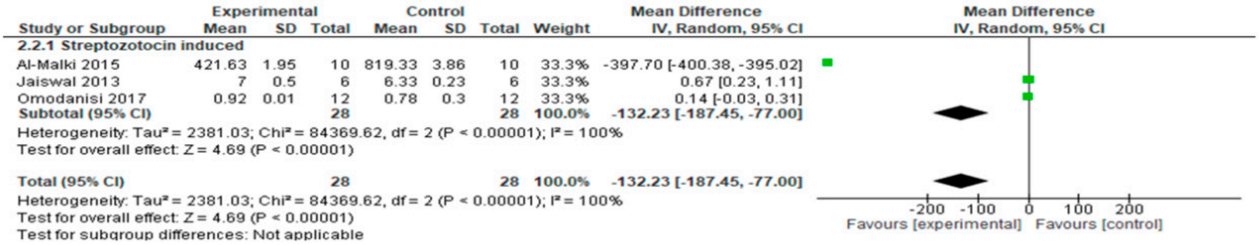

Figure S8. Results of meta-analysis of oxidative status parameters for *Moringa oleifera* extracts: A) Changes in CAT activity, B) Changes in SOD activity.
